# Supplementary material for: Rli51 Attenuates Transcription of the Listeria Pathogenicity Island 1 Gene mpl and Functions as a Trans-Acting sRNA in Intracellular Bacteria
Source: Int J Mol Sci. 2024 Aug 29;25(17):9380. doi: 10.3390/ijms25179380 (PMC11394854; doi:10.3390/ijms25179380)
Supplement: Supplementary file 1 [file ijms-25-09380-s001.zip › Supp-Mat_Moron_24_Final.pdf]

Rli51 attenuates transcription of the *Listeria* pathogenicity island 1 gene *mpl* and functions as a *trans*-acting sRNA in intracellular bacteria

Álvaro Morón-García, Laura Ortiz-Miravalles, Marcos Peñalver-Medina, Francisco García-del Portillo, María Graciela Pucciarelli, and Álvaro Darío Ortega

Supplementary material

Supplementary Figure S1. The secondary structure of Rli51 is not altered upon deletion of ssRNA segments L1, L2.. 2

Supplementary Figure S2. Rli51 functions as a *trans*-acting sRNA in intracellular bacteria...3

Supplementary Figure S3. *rli51* deletion in intracellular bacteria affects the expression of nucleotide-binding proteins, genes involved protein biosynthesis, cell envelope and response to iron...5

Supplementary Table S6. Plasmid constructs generated in this work.........................7

Supplementary Table S7. List of primers used in qPCR assays...................................9

Supplementary Figure S1. The secondary structure of Rli51 is not altered upon deletion of ssRNA segments L1, L2.

Secondary structure prediction of the terminator fold Rli51 and ΔL1 and ΔL2 deletion mutants as determined with PASIFIC webserver. Terminator sequence is highlighted in green and blue. The sequence that hybridizes with the terminator is highlighted in orange and red. ssRNA segments L1 and L2 are indicated with a green and a red box, respectively. Note that the terminator stem loop structure is maintained in both in ΔL1 and ΔL2. Therefore, increased transcriptional read-through observed in translational fusions including ΔL1 and ΔL2 versions of Rli51 does not result from structural changes intrinsic to the genetic modification but from factors that potentially bind the deleted region.

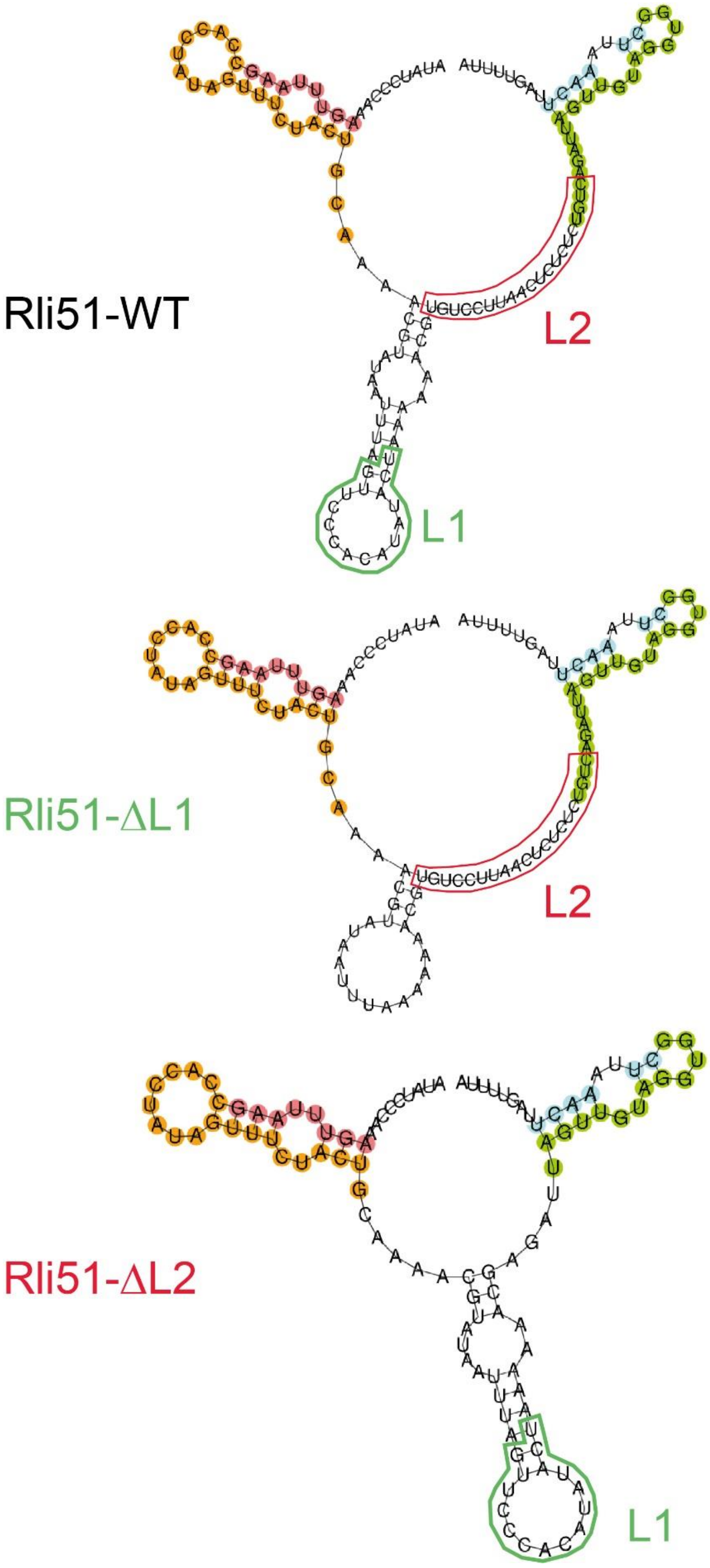

Supplementary Figure S2. Rli51 functions as a *trans*-acting sRNA in intracellular bacteria.

Comparative transcriptomics of intracellular Δ*rli51* *vs.* EGD-e *L. monocytogenes* indicates that deletion of *rli51* sRNA affects the expression of multiple genes.

(a) Volcano plot showing the number of differentially-expressed genes (DEGs) as a result of *rli51* deletion at a glance. X-axis represents differences in expression levels as the Log_2-tranformed value of the fold-change of each gene (ratio of the expression in Δ*rli51* and EGD-e). Y- axis represents statistical significance as the – Log_{10}-tranformed of the p-value. Dotted lines indicate the thresholds used to determine the set of DEGs which will be employed for subsequent functional enrichment and protein-protein interaction network analyses (Supplementary Figure S3). DEGs: $p\text{-val} < 0.05$ and $\text{fold-change} > 1$ (box inset). Green points: down-regulated genes Δ*rli51*; red points: up-regulated genes in Δ*rli51*.

(b) Heatmap showing the magnitude of expression changes of the transcriptome in each sample. Individual genes are indicated in rows. Biological replicates of Δ*rli51* and EGD-e *L. monocytogenes* strains collected from infected JEG-3 cells are indicated in columns. Expression values for each specific gene are represented by color intensity and correspond to the Log_2-tranformed value of the FPKMs in each particular sample normalized by the mean of the FPKMs for that gene in all four samples. Color-intensity scale is indicated on the right.

(c) Principal-component analysis using the FPKMs of all genes in all four samples. The percentage of the variation in the data explained by each dimension is indicated in brackets.

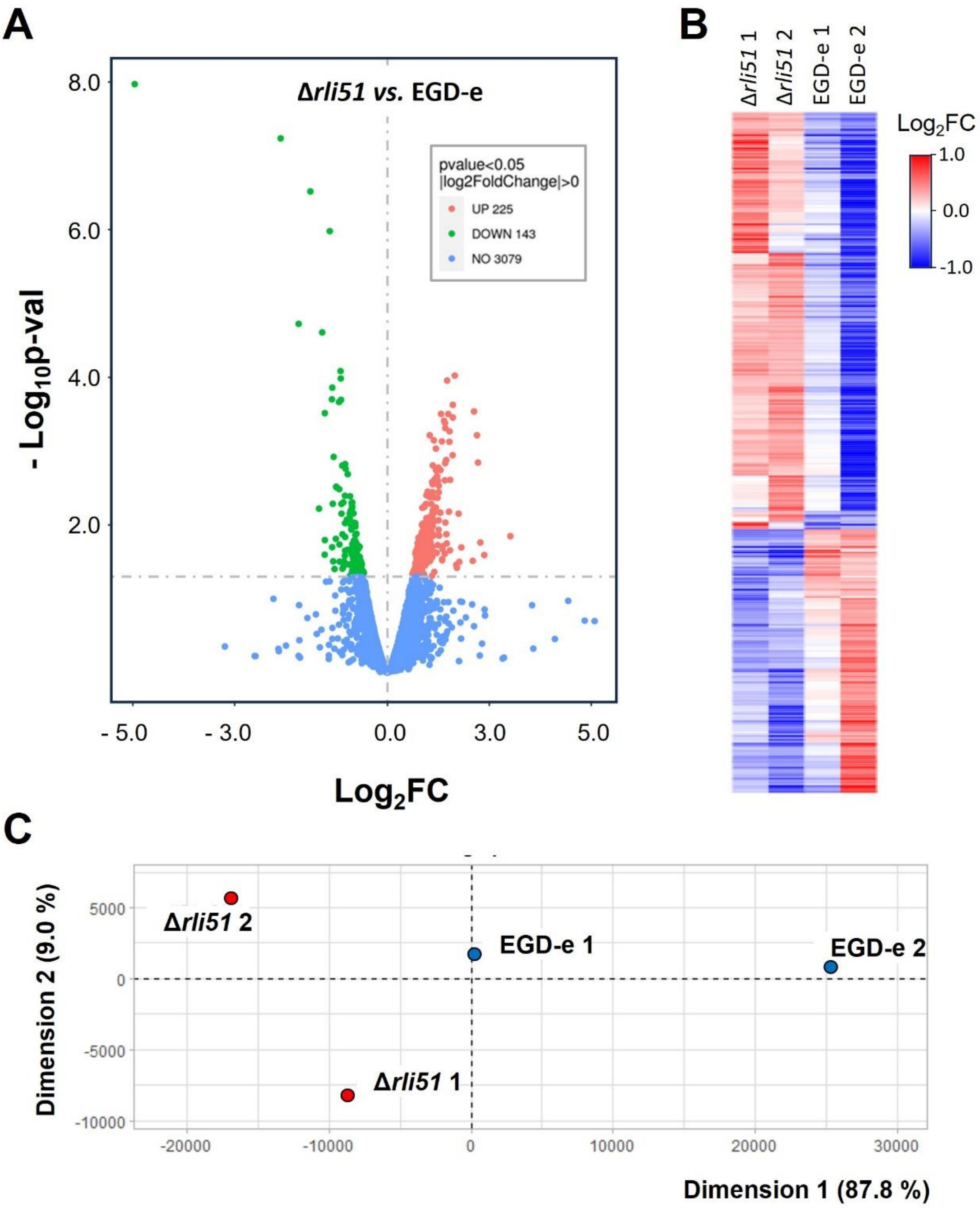
A
Δrli51 vs. EGD-e
pvalue<0.05
|log2FoldChange|>0
UP 225
DOWN 143
NO 3079
- Log10p-val
Log2FC
B
Δrli51 1
Δrli51 2
EGD-e 1
EGD-e 2
Log2FC
1.0
0.0
-1.0
C
Δrli51 2
EGD-e 1
EGD-e 2
Δrli51 1
Dimension 2 (9.0 %)
Dimension 1 (87.8 %)

Supplementary Figure S3. *rli51* deletion in intracellular bacteria affects the expression of nucleotide-binding proteins, genes involved protein biosynthesis, cell envelope and response to iron.

(a) The set of differentially-expressed genes (DEGs) determined as described in Supplementary Figure S2A was used to carry out a functional enrichment analysis based on GO terms. Functions (GO terms) that significantly correlate with up-regulated genes in intracellular Δ*rli51* (Up) are indicated with a point on the upper row while those with down-regulated genes (Down) are on the lower row. The diameter of the points correlates with the number of DEGs assigned to that GO term. The color and intensity of the points correlate with the significance of the result of the analysis. Color and size scales used as reference are indicated below the plot. See Supplementary Table S5 to learn about the set of genes included in each functional group and for further details.

(b) Functional protein association (or protein-protein interaction PPI) networks of DEGs (Supplementary Figure S2A) established with STRING database. The circles (nodes) in the network correspond to proteins and the lines (edges) correspond to high-confidence functional associations. Blue and red colors indicate that the protein is down- or up-regulated in intracellular Δ*rli51*, respectively. The intensity of the color correlates with the magnitude of expression change. Color and intensity scale used as reference is indicated at the bottom right side of the network. See Supplementary Table S6 for more details.

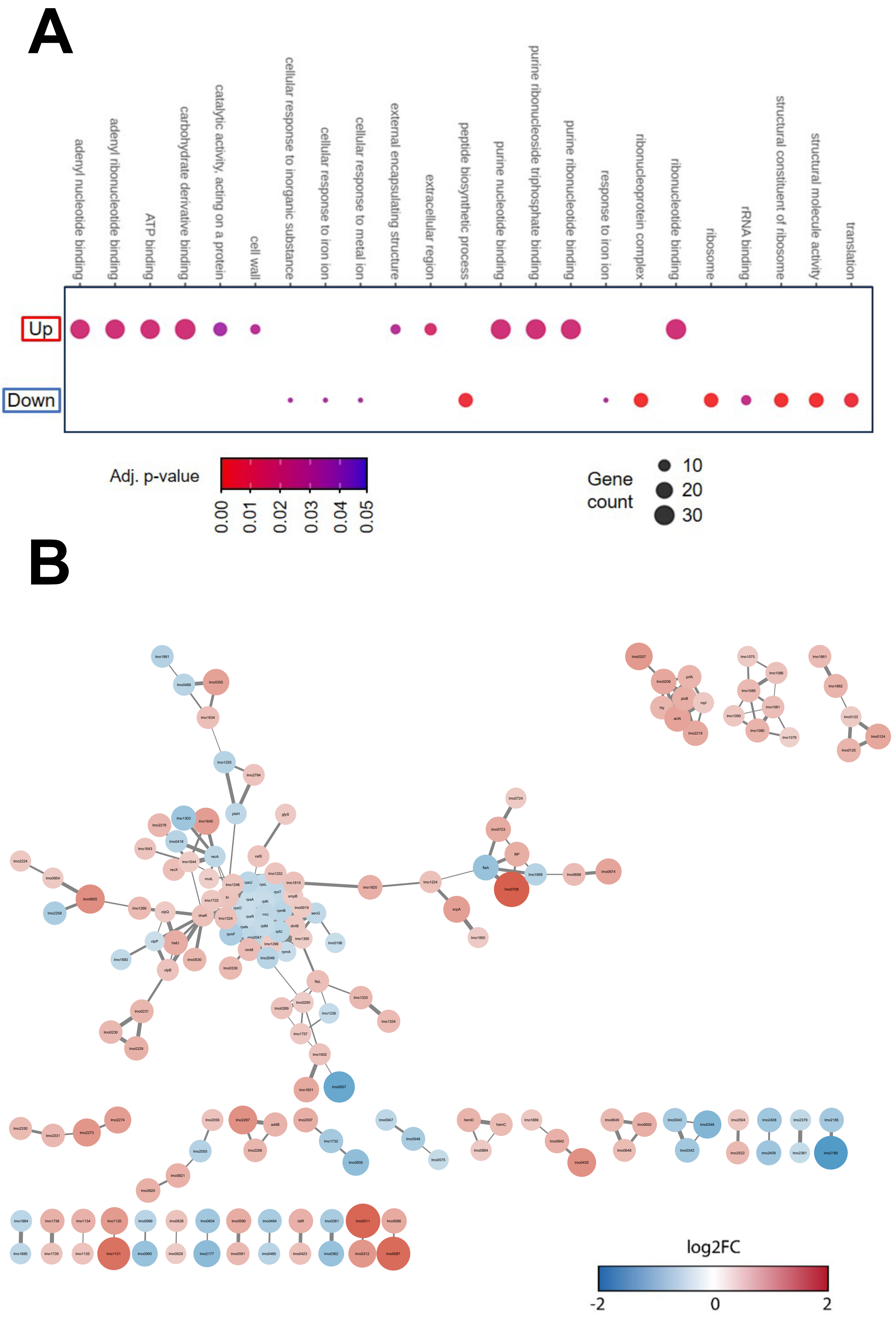
A
adenyl nucleotide binding
adenyl ribonucleotide binding
ATP binding
carbohydrate derivative binding
catalytic activity, acting on a protein
cell wall
cellular response to inorganic substance
cellular response to iron ion
cellular response to metal ion
external encapsulating structure
extracellular region
peptide biosynthetic process
purine nucleotide binding
purine ribonucleoside triphosphate binding
purine ribonucleotide binding
response to iron ion
ribonucleoprotein complex
ribonucleotide binding
ribosome
rRNA binding
structural constituent of ribosome
structural molecule activity
translation
Up
Down
Adj. p-value
0.00
0.01
0.02
0.03
0.04
0.05
Gene count
10
20
30
B
log2FC
-2
0
2

Supplementary Table S6. Plasmid constructs generated in this work.

ID	Brief description	Vector	Insert	Method[1]	RS	Oligo type[1] and sequence (5' - 3')	Template
**pADO-001**	pRMC2-rli51	pRMC2	rli51	PCR	KpnI/EcoRV/BglII<br>EcoRI	A: ggggtaccgatatcagatctatatcccaaagtttaagccacc<br>D: ccggaattcaagtttaagccacctacaact KpnI A:	gDNA
**pADO-002**	pRMC2-GFP	pRMC2	GFP	PCR	 EcoRI	attggtaccatgagtaaaggagaagagcttt<br>D: cggaattcttatttgtatagttcatccatgc	GFP
**pADO-003**	C#1:Rli51-mpl::GFP	pRMC2-GFP	*rli51* promoter-*rli51* -SPACER- 45 nucleotides from *mpl*	PCR	SalI<br>KpnI	A: acgcgtcgactaagaataaaaccgcttaacacaca<br>D: ccggtaccctgaaaagctattaccatgatgat SalI A:	gDNA
**pADO-004**	C#2:ΔPrli51-rli51-mpl::GFP	pRMC2-GFP	*rli51* -SPACER- 45 nucleotides from *mpl*	PCR	 KpnI	acgcgtcgactataagcatatcccaaagtttaagcc<br>D: ccggtaccctgaaaagctattaccatgatgat	gDNA
**pADO-005**	C#3:Phly-5UTRhly-rli5145nt-mpl::GFP	pRMC2	*hly* promoter-5'UTR *hly* -SPACER- 45 nucleotides from *mpl* -GFP	SOE-PCR	SalI<br>-<br>- 	A: acgcgtcgacttgaggcattaacatttgttaacg<br>B: ccttttttaattcgtaaaacttctacattttttaacctaataatgcca<br>C: gtagaagttttacgaattaaaaaggagcg EcoRI D:<br>cggaattcttatttgtatagttcatccatgc	gDNA<br>pADO-003
**pADO-006**	C#4:rli51-hly17nt-mpl::GFP	pRMC2	*rli51* promoter-*rli51* -17 nucleotides from 5'UTR *hly* - 45 nucleotides from *mpl* -GFP	SOE-PCR	SalI<br>-<br>- 	A: acgcgtcgactaagaataaaaccgcttaacacaca<br>B: tctacatttttaacctaagtttaagccacctacaactaatctga<br>C: aggttaaaaaatgtagaaggagcggtgaaatgaaaagtaaac EcoRI<br>D: cggaattcttatttgtatagttcatccatgc	pADO-003<br>pADO-003
**pADO-007**	C#5:5UTRhly-rli5145nt-mpl::GFP	pRMC2	*rli51* promoter- 5'UTR *hly* -SPACER-45 nucleotides from *mpl* -GFP.	SOE-PCR	SalI<br>-<br>- 	A: acgcgtcgactaagaataaaaccgcttaacacaca<br>B: tatatgcttgctttatgcttatattatatggataaacagtcagatattc<br>C: aagcataaagcaagcatataatattgcgtttcatctt EcoRI D:<br>cggaattcttatttgtatagttcatccatgc	pADO-003<br>pADO-005
**pADO-009**	C#7:rli51ΔL2-mpl::GFP	pRMC2	Same as for pADO-003 but with deletion of L2 in rli51: 75 to 95	SOE-PCR	SalI<br>-<br>- 	A: acgcgtcgactaagaataaaaccgcttaacacaca<br>B: ctacaactaatctcgtttttttagtatatgtgggaac<br>C: atactaaaaaacgagattagttgtaggtggcttaa EcoRI D:<br>cggaattcttatttgtatagttcatccatgc	pADO-003<br>pADO-003
**pADO-010**	C#8:rli51Δter-mpl::GFP	pRMC2	Same as for pADO-003 but with deletion of predicted terminator in *rli51:* 105 to 121 and 7 nucleotides of the SPACER.	SOE-PCR	SalI<br>-<br><br>EcoRI	A: acgcgtcgactaagaataaaaccgcttaacacaca<br>B: ctttttaattcgcaactaatctgacagagagagtt - C:<br>gtcagattagttgcgaattaaaaaggagcggtg<br>D: cggaattcttatttgtatagttcatccatgc	pADO-003<br>pADO-003
**pADO-011**	C#9:rli51ΔL1-mpl::GFP	pRMC2	Same as for pADO-003 but with deletion of L1 in *rli51:* 52 to 67	SOE-PCR	SalI<br><br>-<br>EcoRI	A: acgcgtcgactaagaataaaaccgcttaacacaca - B:<br>cacgttttttaaattatacgttttgcagtagaaactat<br>C: gcaaaacgtataatttaaaaaaacgtgtccttaactctc<br>D: cggaattcttatttgtatagttcatccatgc	pADO-003<br>pADO-003
**pADO-012**	Ptet-Δrli51-rli5145nt-mpl::GFP	pRMC2	SPACER-45 nucleotides of *mpl* -GFP	PCR	BglII<br>EcoRI	A: gaagatctagttttacgaattaaaaaggagcgg<br>D: cggaattcttatttgtatagttcatccatgc	pADO-003
**pADO-013**	Ptet-rli51-mpl::GFP	pRMC2	*rli51* -SPACER-45 nucleotides of *mpl* -GFP	PCR	BglII<br>EcoRI	A: gaagatcttataagcatatcccaaagtttaagcc<br>D: cggaattcttatttgtatagttcatccatgc	pADO-003
**pADO-014**	Ptet-5UTRhly-rli5145nt-mpl::GFP	pRMC2	5'UTR *hly* -SPACER-45 nucleotides of *mpl* -GFP	PCR	BglII<br>EcoRI	A: gaagatctataaagcaagcatataatattgcgtttc<br>D: cggaattcttatttgtatagttcatccatgc	pADO-005
**pADO-015**	pRMC2-2xMS2-rli51	pRMC2-rli51	MS2 motifs	RE digestion	BglII<br>EcoRV	n/a	pUTR-MS2[2]
**pADO-016**	pMAD-rli51	pMAD	Upstream (primers A and B) and downstream (C and D) homology flanking regions of *rli51*	PCR	BamHI<br>StuI<br>StuI<br>KpnI	A: gcctcgcgtcgggcgatatcggatccacgttgctcaattcaaca<br>B: tcgtaaaactaggcctttgggatatgcttatattatatgga<br>C: catatcccaaaggcctagttttacgaattaaaaaggagc<br>D: gatctatcgatgcatgccatggtaccgaggatgatttctttttctttca	gDNA

[1] See graphical sketch below.

[2] Ortega AD et al. 2010 J Cell Sci 123 (16), 2685-2696. https://doi.org/10.1242/jcs.065920

RS: Restriction site; SOE PCR: Splicing by Overlap-Extension PCR

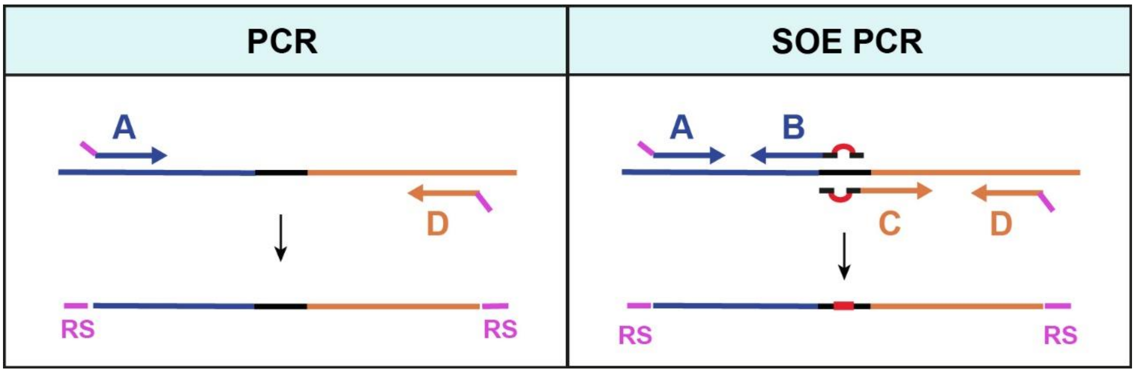

Supplementary Table S7. List of primers used in qPCR assays.

Name	Sequence 5' -3'	Target
**Rli51 qPCR Fw**	GTCAGATTAGTTGTAGGTGGCTTAAACTT	**Rli51-Mpl::GFP (pADO-003)**
**GFP qPCR Rv**	CAGTGAAAAGCTCTTCTCCTTTACTCAT	
**Hly 5UTR qPCR Fw**	GTATTTGGCATTATTAGGTTAAAAAATGTAGA	**hly5UTR-Mpl::GFP (pADO-005, pADO-007)**
**GFP qPCR Rv**	CAGTGAAAAGCTCTTCTCCTTTACTCAT	
**C7 Rli51 qPCR Rw**	AAGTTTAAGCCACCTACAACTAATCT	**Rli51-Mpl-GFP in C#7 (pADO-009)**
**GFP qPCR Rv**	CAGTGAAAAGCTCTTCTCCTTTACTCAT	
**C8 Rli51 qPCR Fw**	TCCTTAACTCTCTCTGTCAGATTAGTTG	**Rli51-Mpl-GFP in C#8 (pADO-010)**
**GFP qPCR Rv**	CAGTGAAAAGCTCTTCTCCTTTACTCAT	
**rli51 Fw**	AAGCCACCTATAGTTTCTACTGCAA	**Rli51**
**rli51 Rw**	GACAGAGAGAGTTAAGGACACGTT	
**GFP qPCR Fw**	TGCGAGATACCCAGATCATATGA	**GFP**
**GFP qPCR Rw**	CTTCGGGCATGGCACTCT	
**rli38 Fw**	AACAAGTTTACTGATTAGGTGCAAA	**Rli38**
**rli38 Rw**	AGCTTTCTATTCCCACTCTGTTTT	
**rpoB Fw**	CGATCTTGGAGAGCCGAAATA	**RpoB**
**rpoB Rw**	GAGCCGCATAGTTTGCATCA	
**lmo1007 Fw**	ATGACTAAACCTGGTAAATACGAAG	**Lmo1007**
**lmo1007 Rw**	GATTGAAGCCTTTAACGCATATTGT	
**lmo2186 Fw**	AATCACTATCCCCTTGTATCACCG	**Lmo2186**
**lmo2186 Rw**	GAAGGGAGATTTAAGAATGAAGAAAGT	
**lmo0937 Fw**	TAATTAATCCACCCGCAATATGG	**Lmo0937**
**lmo0937 Rw**	GCAGAAAGAGGTTTTTATATATGTTAGG	
**Rli123-5'-F**	TGGAAATATAGAGATATCGGCAAGA	**Rli123-L1BS[1]**
**Rli123-5'-R**	TCTGGGCAAATTCACCTACAA	
**Rli123-3'-R**	CCAGCAAATAAACTATTTCGCCATT	**Rli123-L2BS[1]**
**Rli123-3'-F**	AGAGTTACTAAGTTGTATGGGAATCG	
**Rli21-F**	AGAGGTGAGATGACATGTTTCTTT	**Rli21**
**Rli21-R**	GCAGACTTCGCCAATGTAACT	

[1] Rli123 has two predicted binding-sites for Rli51: L1BS (from 49 to 78) and L2BS (from 80 to 94). L1BS is predicted to bind L1 in Rli51, and L2BS is predicted to bind L2 in Rli51. The two pairs of primers were designed such that the amplicons spanned exclusively through each site in Rli123.
